# Application of chimerical ALT perforator flap with vastus lateralis muscle mass for the reconstruction of oral and submandibular defects after radical resection of tongue carcinoma: a retrospective cohort study

**DOI:** 10.1186/s12903-020-01066-x

**Published:** 2020-03-30

**Authors:** Rong Yang, Xiaoshan Wu, Pathak Ajit Kumar, Yafei Xiong, Canhua Jiang, Xinchun Jian, Feng Guo

**Affiliations:** Department of Oral and Maxillofacial Surgery, Xiangya Hospital, Central South University, Changsha, 410008 Hunan province China

**Keywords:** Tongue malignant tumor, Reconstruction, Perforator flap, Chimeric flap

## Abstract

**Background:**

Patients with tongue carcinoma who undergo combined tongue and neck radical resection often have simultaneous oral and submandibular defects. Due to its high flexibility, the anterolateral thigh (ALT) perforator flap is gradually being adopted by surgeons for oral reconstruction. However, the tissue volume of perforator flaps is insufficient for the reconstruction of both the oral and submandibular regions. In this retrospective cohort study, we compared the postoperative outcomes and complications between patients reconstructed with using the classical ALT perforator flap and patients reconstructed using the chimeric ALT perforator flap with vastus lateralis muscle mass.

**Methods:**

From August 2017 to August 2019, 25 patients underwent reconstructive therapy using a classical ALT perforator flap (classical group), while 26 patients were reconstructed with the chimeric ALT perforator flap (chimeric group) after radical resection of tongue cancer in Xiangya Hospital, Central South University. The flap survival rate, incidence of submandibular infection, lateral appearance, lower extremity function, and quality of life were compared between the two groups.

**Results:**

There were no differences in flap survival rate and postoperative lower extremity function between the two groups. The incidence of submandibular infection was 15.4 and 40% in the chimeric and classical group, respectively. The duration of recovery was 12.20 ± 2.69 and 15.67 ± 4.09 days in the chimeric and classical group, respectively. The submandibular region fullness was satisfactory in the chimeric group. The postoperative quality of life in the chimeric group was better than that in the classical group (*P <* 0.05).

**Conclusions:**

The chimerical ALT perforator flap with muscle mass reconstructs both the oral and submandibular defects accurately. It maintains the profile and fullness of the submandibular region and may reduce the incidence of submandibular infection.

## Background

Oral squamous cell carcinoma (OSCC) is one of the most common malignant tumors in the body, with tongue cancer as the most frequently observed type of OSCC. It has been reported that the 5-year survival rate of tongue cancer is about 60%. The incidence rates of mortality and recurrence have increased in recent years due to local recurrence and submandibular cervical lymph node metastasis [1–3]. Therefore, it is an important challenge for surgeons to develop reasonable surgical treatment strategies, including therapeutic resection, postoperative defect reconstruction, and functional restoration.

Treatment with surgical resection, adjuvant radiotherapy, and chemotherapy are the main therapeutic strategies for the tongue carcinoma. The purpose of surgical treatment is to excise the primary tumor with a wide margin. The tongue is a complex organ which is composed of striated muscles. The tumor cells often migrate from the primary site, infiltrate into the muscle, and develop into local recurrences in the mouth floor and submandibular region. Because of the special anatomical features of the tongue, patients with tongue carcinoma often experience early lymph node metastasis [4–6]. Therefore, the thoroughness of the resection in the case of tongue cancer is of particularly importance. It has been recently suggested that the upper lingual muscle groups, such as the genioglossus muscle, geniohyoid muscle, mylohyoid muscle, and/or anterior belly of digastric muscle should also be resected in the radical resection to reduce the possibility of recurrence [5, 7, 8]. However, defects in the mouth floor and submandibular region post-operation result in the creation of a large unused space, leading to complications such as submandibular wound infection and oral fistula, thereby prolonging the recovery period of the patients and seriously affecting their prognosis [7, 9].

It is challenging to reconstruct the oral and submandibular defects simultaneously [10, 11]. In recent decades, free anterolateral thigh flap (ALT) has become one of the main choices for reconstruction due to its high reliability and versatility [11]. The traditional ALT carries redundant subcutaneous tissue, fascia lata, and muscle, with a high volume of tissue volume that is far beyond the needs of the surgery. As a result, the perforator ALT flap was developed. The vascular pedicle was skeletonized to obtain the ideal length and to avoid the use of redundant amounts of tissue, which allowed for the accurate repair of the primary oral defect [12–15]. However, the defect of submandibular region is left unfilled, which may lead to the infection and depression of the submandibular region. To address these shortcomings, the perforator-based chimeric flap with a muscle component has been applied in the reconstruction of complex extremity defects [16]. However, it has not yet been used for the reconstruction of both the oral and submandibular defects after radical resection of tongue cancer.

In this retrospective cohort study, the flap survival rate, the duration of flap harvesting, the incidence of submandibular infection, and lateral appearance were compared between two groups treated with different reconstruction methods. The first group underwent reconstructive therapy with a classical perforator ALT flap, while the other group underwent reconstructive therapy with the chimeric perforator ALT flap, with the muscle mass at the end of the descending branch of the lateral circumflex femoral artery (LCFA).

## Methods

### Study population

A total of 51 patients with primary tongue carcinoma who had previously undergone extensive surgical resection in the Department of Oral and Maxillofacial Surgery, Xiangya Hospital of Central South University between August 2017 and August 2019 were enrolled in this retrospective study. All patients had undergone radical tumor resection. The genioglossus muscle, geniohyoid muscle, mylohyoid muscle, and anterior belly of the digastric muscle were resected for all patients due to aggressive tumor invasion. The Medical Ethics Committee of Xiangya Hospital, Central South University approved this study. The patient’s identity was preserved.

Demographic information on the patients, including age, sex, tumor stage, pathological T or N status, size of dead cavity, and size of flap, were collected and analyzed [17]. The inclusion criteria were as follows: (1) previously untreated oral cancer; (2) pathologically proven as squamous cell carcinoma of tongue by biopsy before surgery; (3) reconstruction with ALT perforator flap or ALT chimeric flap. The exclusion criteria included: (1) history of previous craniofacial surgery; (2) distant metastasis or contraindication for curative surgery; (3) the postoperative follow-up data were incomplete or lost; (4) patients who received radiotherapy, chemotherapy, and other treatment before operation.

Among these patients, 25 cases underwent reconstructive therapy using the classical ALT perforator (classical group), while 26 cases underwent reconstructive therapy using the chimerical ALT perforator flap with a mass of vastus lateralis muscle on the distal runoff of the lateral circumflex femoral artery (chimeric group). For all cases, the resection of the primary sites and reconstruction were performed by one surgeon (Dr. Feng Guo) and his team. All of the cases underwent wound recovery assessment and management during the period between the date of surgery and the date of wound healing. The follow-up duration was 6 to 24 months.

### Harvesting of the classical and chimerical ALT perforator flap

**Fig. 1:**
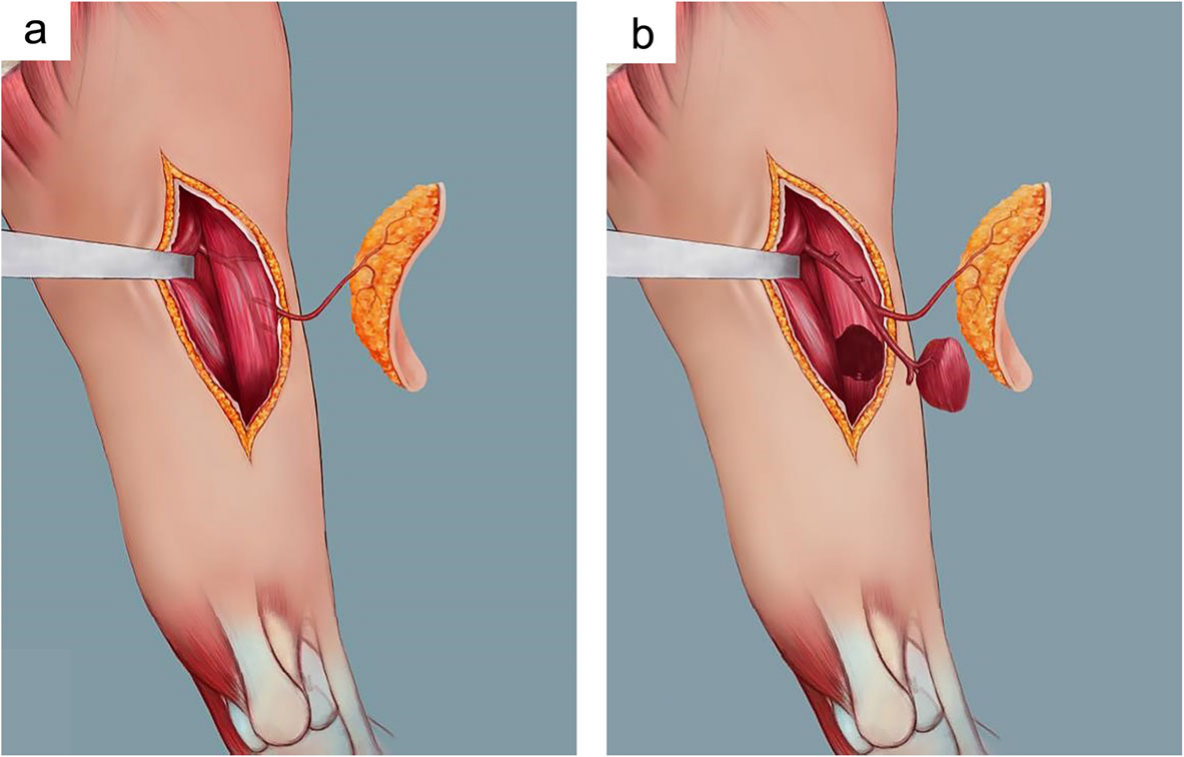
Schematic representation of the chimeric and classical ALT flaps. **a** Classical ALT perforator flap of descending branch of lateral circumflex femoral artery. **b** Chimeric ALT flap with vastus lateralis muscle mass

For the harvesting of the classical ALT perforator flap, we used a previously described method [18]. Briefly, doppler ultrasonography was used to map the perforator in the anterolateral thigh region prior to surgery. After radical resection, we dissected the pre-positioned perforator to the descending branch of the LCFA retrogradely without destroying the integrity of the fascia lata (Fig. 1a).

**Fig. 2:**
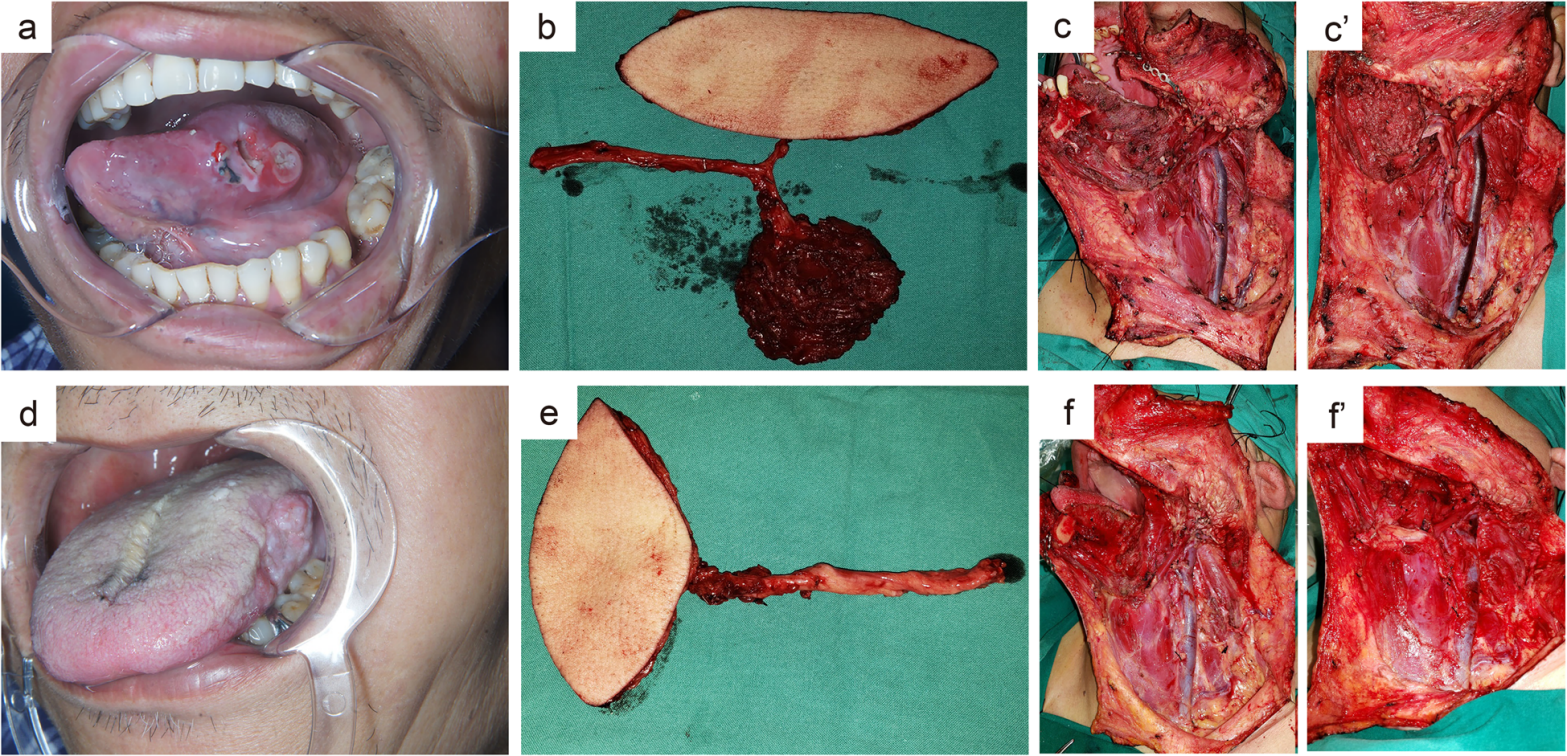
Comparison of the reconstruction of oral and submandibular defects using the two types of flaps. **a**-**c**. Reconstruction of oral and submandibular defects using the chimeric ALT flap: (**a**) the size and location of primary tumor; (**b**) the chimeric ALT perforator flap; (**c, c’**) oral and submandibular defects before and after transplantation with the chimeric ALT perforator flap. **d**-**f**. Reconstruction of the oral and submandibular defects using the classical ALT flap: (**d**) the size and location of the primary tumor; (**e**) the classical ALT perforator flap; (**f, f’**) oral and submandibular defects before and after transplantation with the classical ALT perforator flap

For the chimeric flap, the vastus lateralis muscle corresponding to the size of the dead space was harvested at the distal end of the descending branch of the LCFA. The vessel distance between the distal end of muscle mass and the perforator branch was determined by the distance between the defect of oral mucosa and the submandibular dead space (Fig. 1b). In the reconstruction, the flap was used to repair the defect of oral cavity, and the muscle mass was used to repair the submandibular dead space (Fig. 2).

In this study, free flap failure was defined when the flap was completely or partially lost, that is when a large enough area of the flap was lost that prevented obtaining the intended functional results [19].

### Assessment of donor site function and quality of life

The Lower Extremity Functional Scale (LEFS) was used to assess the donor site function [20]. It contains 20 items, each of which is rated on a 5-point scale, from 0 (extreme difficulty or unable to perform) to 4 (no difficulty). The 20 items are listed in the Additional file 1.

The University of Washington Quality of Life Scale (UW-QoL, Version 4) was used to record health-related quality of life in patients with head and neck cancer. It contains 12 questions referring to pain, appearance, activity, recreation, swallowing, chewing, speech, shoulder function, taste, saliva, mood, and anxiety. The answer to each question is scored from 0 to 100 [21]. Detailed information on the UW-QoL is provided in Additional file 2.

### Data collection and analysis

Surgical parameters, including the duration of harvesting flap, the survival rate of the flap, the incidence of submandibular infection, and the duration of recovery, were collected. The function of the donor area, the appearance of the submandibular region, and the quality of life were recorded and analyzed after patient recovery. Data were compared between the two groups using Fisher’s accurate test and unpaired Student’s *t*-test (SPSS 22.0). *P* < 0.05 was considered statistically significant.

## Results

### Patient demographics

The mean age of the chimeric flap group was 51.73 ± 8.42 years, and the classical ALT flap group was 48.41 ± 7.83 years. The clinical stages and the metastasis of lymph nodes were compared between two groups, and no statistically significant difference was found (Table 1).

**Table 1:** Characteristics of the patients reconstructed with chimeric flap and classical flap

Characteristic	Flap		*P*
	Chimeric flap (*n* = 26)	Classical flap (*n* = 25)	
Age, years (mean ± SD)	51.73 ± 8.42	48.24 ± 7.83	0.14
Sex, male: female, n: n	25:1	24:1	0.98
Tumor stage			0.42
T1	4	6	
T2	20	16	
T3	1	3	
T4	1	0	
Nodal stage			0.54
N0	16	13	
N1	6	4	
N2	2	5	
N3	2	3	
Clinical stage			0.78
I	4	3	
II	11	9	
III	6	5	
IV	5	8	
Size of flap (range)	8 cm × 4.5 cm–14 cm × 6 cm	7 cm × 4 cm–14 cm × 7 cm	0.60
Size of muscle (range)	3 cm × 3 cm–5 cm × 4 cm	–	
Size of dead cavity (range)	3 cm × 3 cm–5 cm × 4 cm	3 cm × 3 cm–5 cm × 4 cm	0.34

Date presented as mean ± SD or n, unless otherwise indicated

### Flap harvesting time and survival rate

The flap harvesting time was 99.12 ± 28.30 min in the chimeric group, and 96.71 ± 20.64 min in the classical group. There was no significant difference between the two groups (*P* = 0.92). The success rate of the flap was 100% in the chimeric group, and 92% in the classical group. A partial loss of the flap was observed in two patients in the classical group. There was no significant difference between the groups (*P* = 0.98) (Table 2).

**Table 2:** Comparison of indicators in the recovery period between the chimeric group and classical groups

Indicators		Chimeric group (*n* = 26)	Classical group (*n* = 25)	*P*
Submandibular wound infection (rate)	A	4/26	5/25	0.025*
	B	0	3/25	
	C	0	2/25	
Wound healing time (days)		12.20 ± 2.69	15.67 ± 4.09	0.0054*
Flap operation time (min)		99.12 ± 28.30	96.71 ± 20.64	0.92
Flap survival rate				0.98
Success		26/26	23/25	
Partial loss		0	2/25	

A: submandibular wound skin redness and edema; B: submandibular wound skin redness with a fluctuant swelling; C: sub oral fistula. Data presented as mean ± SD or n/n, unless otherwise indicated. *Statistically significant difference

### Postoperative complications and recovery time

Since submandibular infection is the main postoperative complication of this type of surgery, it was compared between the two treatment groups. Among the 26 patients in the chimeric group, 4 cases experienced submandibular skin redness and edema. Early interventions, including local drainage, compression bandage, and antibiotics treatment, were performed to promote healing. The other 22 patients healed in the first stage. The rate of the submandibular infection was 15.4%.

Among the 25 patients in the classical group, 5 cases experienced submandibular skin redness and edema. Three cases also had fluctuant swelling in the submandibular region. Two cases developed submandibular fistula. Treatment with antibiotics, local effective drainage, and compression bandage were used for these 10 cases. The two patients with submandibular fistula were treated with a second operation for intraoral and wound debridement and closure. In total, 10 cases developed submandibular infection (40%). There was a significant difference between the chimeric and classical groups in terms of the rate of submandibular infection (*P =* 0.025).

The recovery time from the first surgical procedure to rehabilitation was 12.20 ± 2.69 days for the chimeric group and 15.67 ± 4.09 days for the classical group. This difference was statistically significant (*P* = 0.0054).

Our findings indicate that the chimeric ALT flap can significantly reduce the incidence of submandibular wound infection and shorten the duration of wound healing.

### Risk factors for wound infection

To identify risk factors for wound infection, a univariate analysis of risk factors was performed. The results showed statistically significant differences for the risk factors of smoking, betel nut consumption, diabetes, high blood pressure (HBP), and type of flap between the infection and non-infection groups (*P* < 0.05) (Table 3).

**Table 3:** Univariate analysis of the risk factors for wound infection

	Groups			
Variables	Infection	Non-infection	X^2^	*P*
Age (years)			1.902	0.1679
< 50	1	9		
> 50	13	28		
Tumor stage			2.949	0.0859
T1-T2	11	35		
T3-T4	3	2		
Nodal stage			0.559	0.4547
N0-N1	10	30		
N2-N3	4	7		
Clinical stage			1.131	0.2876
I-II	6	22		
III-IV	8	15		
Cigarette			3.996	0.0456*
No	1	13		
Yes	13	24		
Alcohol			2.695	0.1006
No	3	2		
Yes	11	35		
Betel nut			4.135	0.0420*
No	0	9		
Yes	14	28		
Diabetes			4.006	0.0453*
No	8	31		
Yes	6	6		
HBP			5.251	0.0219*
No	10	35		
Yes	4	2		
Type of flaps			3.878	0.0489*
Chimeric flap	4	22		
Classical flap	10	15		

The chi-square test was used. *P* < 0.05 was considered to be statistically significant. *HBP* high blood pressure. *Statistically significant difference

We then analyzed the correlation of these factors with wound infection using multivariate logistic regression analysis. The results showed that the risk factors for wound infection were HBP (odds ratio [OR] = 34.048; 95% CI (2.051–565.264); *P* = 0.014), and type of flaps (OR = 19.258; 95% CI (1.940–191.150); *P* = 0.012) (Table 4).

**Table 4:** Multivariate logistic regression analysis for the risk factors of wound infection

Variables	Wals	OR (95%CI)	*P*
cigarette	–	–	NS
Betel nut	–	–	NS
Diabetes	–	–	NS
HBP	6.057	34.048 (2.051–565.264)	0.014*
Type of flaps	6.381	19.258 (1.940–191.150)	0.012*

*P* < 0.05 was considered to be statistically significant. *HBP* high blood pressure. *Statistically significant difference

### Submandibular appearance

**Fig. 3:**
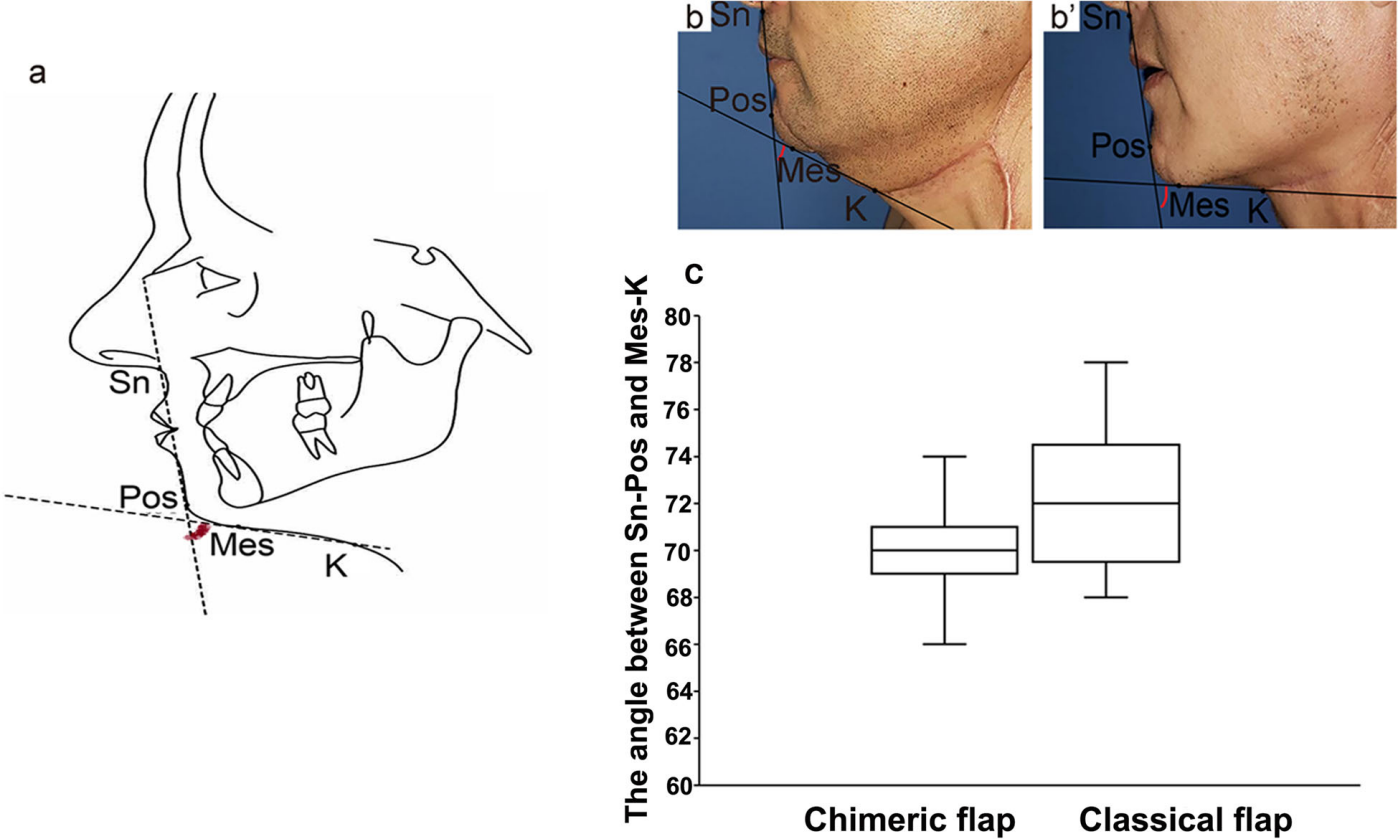
Comparison of submandibular appearance between the two treatment groups. **a** Assessment of submandibular appearance by measuring the angle between Sn-Pos and K-Pos. **b** Submandibular appearance of tongue cancer patients after chimeric ALT perforator flap reconstruction. **b’** Submandibular appearance of tongue cancer patients after classical ALT perforator flap reconstruction. **c** Statistical results of the comparison between the two groups

We evaluated the appearance of the submandibular region after surgery by measuring the angle between Sn-Pos and Mes-K in lateral profile photos (Fig. 3a). The results were statistically evaluated and compared between the two groups. The angle of the chimeric group was 70.19 ± 2.304, while that of the classical group was 72.00 ± 3.072. There was a significant difference between the two groups (*P* = 0.0236) (Fig. 3b, c).

### Donor site function and quality of life post-operation

**Fig. 4:**
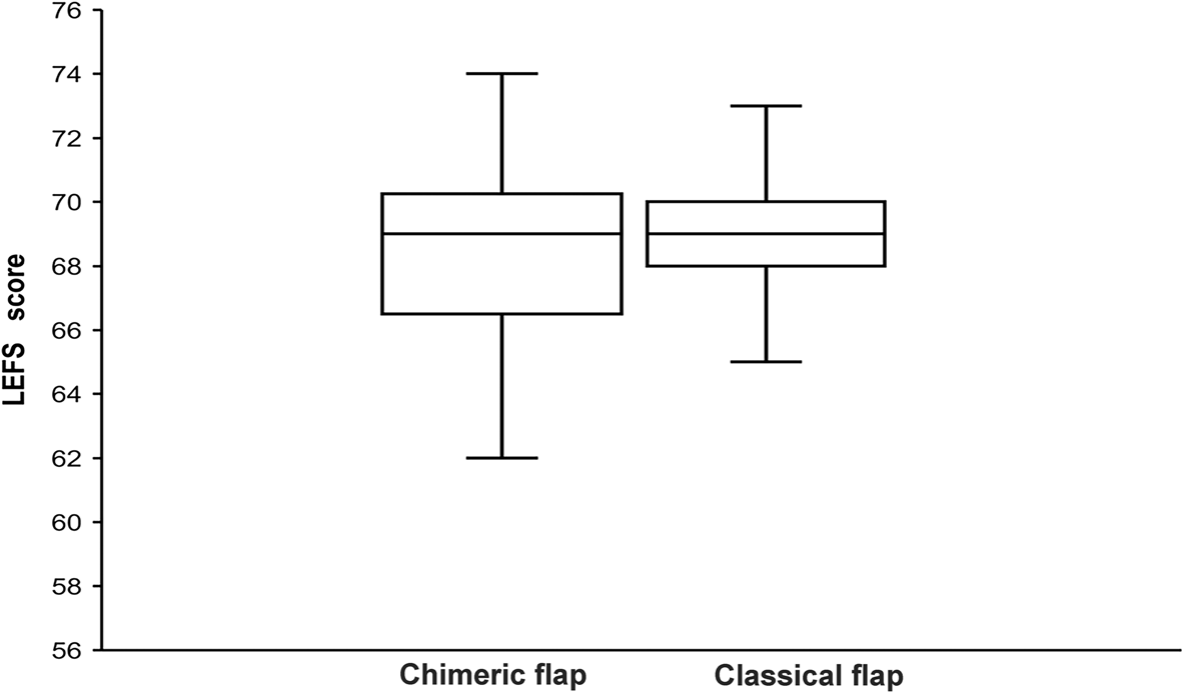
Bar graph illustrating the differences in LEFS between the two groups treated with chimeric and classical flaps

The Lower Extremity Functional Scale (LEFS) [20] was used to assess the 51 patients six months after the operation (Additional file 1). The results were statistically evaluated. The LEFS score was 68.28 ± 2.95 in the chimeric group and 68.89 ± 2.30 in the classical group, and there was no statistical difference (*P* = 0.483). These findings showed that the function of the donor site was not significantly affected in the group treated with the chimeric ALT perforator flap (Fig. 4).

A UW-QOL questionnaire [21] was used to assess the patients six months after the operation (Additional file 2). The results showed that the value of mastication in the chimeric group was higher than that in the classical group (*P* = 0.032). The other items were similar between the two groups (Table 5).

**Table 5:** Comparison of UW-QOL scores between the chimeric group and classical groups

Indicators	Chimeric group	Classical group	*P*
Pain	70.65 ± 25.22	69.60 ± 25.77	0.89
Activity	66.15 ± 19.32	63.00 ± 20.49	0.67
Recreation	64.42 ± 23.18	61.80 ± 24.85	0.70
Swallowing	63.65 ± 22.26	54.40 ± 23.04	0.16
Chewing	66.92 ± 18.76	54.60 ± 20.39	0.032*
Speech	55.77 ± 18.33	55.00 ± 19.90	0.88
Shoulder	67.88 ± 21.67	69.80 ± 17.63	0.74
Taste	62.12 ± 22.37	57.60 ± 21.73	0.48
Saliva	57.88 ± 20.29	56.80 ± 23.15	0.86
Mood	61.73 ± 25.94	60.80 ± 22.26	0.89
Anxiety	59.62 ± 21.26	57.80 ± 18.55	0.75

*Statistically significant difference

## Discussion

In this retrospective cohort study, we compared the postoperative outcome and complications between the patients reconstructed with the classical ALT perforator flap and the patients reconstructed with a chimeric ALT perforator flap with vastus lateralis muscle mass. The results showed that the infection rate was reduced and the submandibular fullness was satisfactory in the chimeric group.

The process of lymphatic drainage of tongue tissue is rich and complex [1]. Lymphatic fluid first concentrates in the floor of the mouth and is then transferred into the upper cervical lymph nodes [2]. Tumor cells move downwards, along the path of least resistance [22]. The metastasis of the lymph nodes of the floor of the mouth is difficult to observe and diagnose [4]. As a result, it has been proposed that the radical dissection of tongue cancer should contain both the primary lesions and possible pathways of metastasis, including the partial suprahyoid muscle groups and the lymphatic, neurovascular, and glandular tissues [5, 7, 8]. In this study, we resected the primary lesions and partial suprahyoid muscle groups in all cases. However, as a result, an extensive empty space in the floor of the mouth and submandibular region was left.

The reconstruction of both the primary site and the floor of the mouth for tongue cancer patients is a challenge for surgeons. The anterolateral thigh flap (ALT) has been used for reconstruction in tongue cancer patients for several decades [8, 12, 13]. However, perforators are not dissected through vastus lateralis muscle in the traditional ALT flap and thus the muscle and overlying skin are generally harvested as a single, large section [12, 13]. Because of the poor flexibility of the traditional ALT flap, the shape of the reconstructed tongue is generally unsatisfactory. In this study, the perforator was dissected through the muscle, and the vastus lateralis muscle component was carried in the distal runoff of the descending branch of LCFA in the chimeric group [23]. We found that the chimeric perforator ALT flap with a muscle component was suitable for most cases of tongue cancer, with the following advantages: (1) The damage of donor site is reduced as much as possible because both the flap and muscle component are harvested precisely according to the size of oral and submandibular defects, which obeys the principle of “economy of donor site incisions” [16, 24]; (2) The distance between the perforator of the flap and the muscle component is about 3–5 cm, which provides a greater degree of freedom to inset the muscle mass [23, 25].

The perforator-based chimeric flap has been used in the reconstruction of extensive extremity defects with a deep and slender dead space [23]. Recently, it has also been used in complex or multiple defects in the head and neck region [26]. For example, Lai et al. [27] used the chimeric ALT flap to reconstruct the complete loss of upper and lower lips. Zeng et al. [28] used the chimeric ALT perforator flap to reconstruct the complex total parotidectomy defect. Jiang et al. [29] used multipaddled ALT flaps to reconstruct multiple oral defects. However, this study is the first to report on the application of the chimeric ALT perforator flap with a muscle component after the radical resection of tongue cancer. We found that the chimeric ALT perforator flap could be used to significantly reduce the incidence of wound infection, shorten the time of wound healing, and obtain a better submandibular appearance.

In this retrospective study, the factors of HBP and the types of flap used were found to have a significant effect on the rate of wound infection, using both univariate and multivariate analyses. However, other risk factors, including smoking, betel nut consumption, and diabetes, were only found to be significant in the univariate analysis, and not in the multivariate analysis, which may result from an insufficient sample size. Therefore, a prospective case-control study with a larger sample size will be needed to verify our findings.

## Conclusion

The chimerical ALT perforator flap with vastus lateralis muscle mass can be used to accurately and safely reconstruct both oral and submandibular defects. This type of perforator flap maintains the profile and fullness of the submandibular region and may reduce the incidence of submandibular infection.

## Supplementary information

**Additional file 1.** Lower Extremity Functional Scale (LEFS).

**Additional file 2.** University of Washington Quality of Life Questionnaire.

## Data Availability

The data sets used and/or analyzed in this study are available from the corresponding author upon reasonable request.

## Abbreviations

ALT: Anterolateral thigh cutaneous
OSCC: Oral squamous cell carcinoma
LEFS: The Lower Extremity Functional Scale
UW-QOL: The University of Washington quality of Life questionnaire
TNM: Tumor, node, metastasis
LCFA: Lateral circumflex femoral artery
HBP: High blood pressure
