# Supplementary material for: Application of chimerical ALT perforator flap with vastus lateralis muscle mass for the reconstruction of oral and submandibular defects after radical resection of tongue carcinoma: a retrospective cohort study
Source: BMC Oral Health. 2020 Mar 30;20:94. doi: 10.1186/s12903-020-01066-x (PMC7106716; doi:10.1186/s12903-020-01066-x)
Supplement: Supplementary file 1 — Additional file 1. Lower Extremity Functional Scale (LEFS). [file 12903_2020_1066_MOESM1_ESM.pdf]

Lower Extremity Functional Scale (LEFS)

Source: Binkley JM, Stratford PW, Lott SA, Riddle DL. The Lower Extremity Functional Scale (LEFS): scale development, measurement properties, and clinical application. North American Orthopaedic Rehabilitation Research Network. *Phys Ther*. 1999 Apr;79(4):371-83.

The Lower Extremity Functional Scale (LEFS) is a questionnaire containing 20 questions about a person's ability to perform everyday tasks. The LEFS can be used by clinicians as a measure of patients' initial function, ongoing progress and outcome, as well as to set functional goals.

The LEFS can be used to evaluate the functional impairment of a patient with a disorder of one or both lower extremities. It can be used to monitor the patient over time and to evaluate the effectiveness of an intervention.

Scoring instructions

The columns on the scale are summed to get a total score. The maximum score is 80.

Interpretation of scores

- The lower the score the greater the disability.
- The minimal detectable change is 9 scale points.
- The minimal clinically important difference is 9 scale points.
- % of maximal function = (LEFS score) / 80 * 100

Performance:

- The potential error at a given point in time was +/- 5.3 scale points.
- Test-retest reliability was 0.94.
- Construct reliability was determined by comparison with the SF-36. The scale was found to be reliable with a sensitivity to change superior to the SF-36.

Instructions

We are interested in knowing whether you are having any difficulty at all with the activities listed below **because of your lower limb problem** for which you are currently seeking attention. Please provide an answer for **each** activity.

Today, *do you* or *would you* have any difficulty at all with:

Activities	Extreme difficulty or unable to perform activity	Quite a bit of difficulty	Moderate difficulty	A little bit of difficulty	No difficulty
1. Any of your usual work, housework or school activities.	0	1	2	3	4
2. Your usual hobbies, recreational or sporting activities.	0	1	2	3	4
3. Getting into or out of the bath.	0	1	2	3	4
4. Walking between rooms.	0	1	2	3	4
5. Putting on your shoes or socks.	0	1	2	3	4
6. Squatting.	0	1	2	3	4
7. Lifting an object, like a bag of groceries from the floor.	0	1	2	3	4
8. Performing light activities around your home.	0	1	2	3	4
9. Performing heavy activities around your home.	0	1	2	3	4
10. Getting into or out of a car.	0	1	2	3	4
11. Walking 2 blocks.	0	1	2	3	4
12. Walking a mile.	0	1	2	3	4
13. Going up or down 10 stairs (about 1 flight of stairs).	0	1	2	3	4
14. Standing for 1 hour.	0	1	2	3	4
15. Sitting for 1 hour.	0	1	2	3	4
16. Running on even ground.	0	1	2	3	4
17. Running on uneven ground.	0	1	2	3	4
18. Making sharp turns while running fast.	0	1	2	3	4
19. Hopping.	0	1	2	3	4
20. Rolling over in bed.	0	1	2	3	4
**Column Totals:**	0	1	2	3	4
