# Supplementary material for: Application of chimerical ALT perforator flap with vastus lateralis muscle mass for the reconstruction of oral and submandibular defects after radical resection of tongue carcinoma: a retrospective cohort study
Source: BMC Oral Health. 2020 Mar 30;20:94. doi: 10.1186/s12903-020-01066-x (PMC7106716; doi:10.1186/s12903-020-01066-x)
Supplement: Supplementary file 2 — Additional file 2. University of Washington Quality of Life Questionnaire. [file 12903_2020_1066_MOESM2_ESM.pdf]

Name: ____________________
Date: ____________________

University of Washington Quality of Life Questionnaire (UW-QOL)

This questionnaire asks about your health and quality of life **over the past seven days**. Please answer all of the questions by checking one box for each question.

1. **Pain**. (Check one box: ☑)

- ☐ I have no pain.
- ☐ There is mild pain not needing medication.
- ☐ I have moderate pain - requires regular medication (codeine or nonnarcotic).
- ☐ I have severe pain controlled only by narcotics.
- ☐ I have severe pain, not controlled by medication.

2. **Appearance**. (Check one box: ☑)

- ☐ There is no change in my appearance.
- ☐ The change in my appearance is minor.
- ☐ My appearance bothers me but I remain active.
- ☐ I feel significantly disfigured and limit my activities due to my appearance.
- ☐ I cannot be with people due to my appearance.

3. **Activity**. (Check one box: ☑)

- ☐ I am as active as I have ever been.
- ☐ There are times when I can't keep up my old pace, but not often.
- ☐ I am often tired and have slowed down my activities although I still get out.
- ☐ I don't go out because I don't have the strength.
- ☐ I am usually in bed or chair and don't leave home.

4. **Recreation**. (Check one box: ☑)

- ☐ There are no limitations to recreation at home or away from home.
- ☐ There are a few things I can't do but I still get out and enjoy life.
- ☐ There are many times when I wish I could get out more, but I'm not up to it.
- ☐ There are severe limitations to what I can do, mostly I stay at home and watch TV.
- ☐ I can't do anything enjoyable.

5. **Swallowing**. (Check one box: ☑)

- ☐ I can swallow as well as ever.
- ☐ I cannot swallow certain solid foods.
- ☐ I can only swallow liquid food.
- ☐ I cannot swallow because it "goes down the wrong way" and chokes me.

6. **Chewing**. (Check one box: ☑)

- ☐ I can chew as well as ever.
- ☐ I can eat soft solids but cannot chew some foods.
- ☐ I cannot even chew soft solids.

©University of Washington, 1999

7. **Speech**. (Check one box: ☑)

- ☐ My speech is the same as always.
- ☐ I have difficulty saying some words but I can be understood over the phone.
- ☐ Only my family and friends can understand me.
- ☐ I cannot be understood.

8. **Shoulder**. (Check one box: ☑)

- ☐ I have no problem with my shoulder.
- ☐ My shoulder is stiff but it has not affected my activity or strength.
- ☐ Pain or weakness in my shoulder has caused me to change my work.
- ☐ I cannot work due to problems with my shoulder.

9. **Taste**. (Check one box: ☑)

- ☐ I can taste food normally.
- ☐ I can taste most foods normally.
- ☐ I can taste some foods.
- ☐ I cannot taste any foods.

10. **Saliva**. (Check one box: ☑)

- ☐ My saliva is of normal consistency.
- ☐ I have less saliva than normal, but it is enough.
- ☐ I have too little saliva.
- ☐ I have no saliva.

11. **Mood**. (Check one box: ☑)

- ☐ My mood is excellent and unaffected by my cancer.
- ☐ My mood is generally good and only occasionally affected by my cancer.
- ☐ I am neither in a good mood nor depressed about my cancer.
- ☐ I am somewhat depressed about my cancer.
- ☐ I am extremely depressed about my cancer.

12. **Anxiety**. (Check one box: ☑)

- ☐ I am not anxious about my cancer.
- ☐ I am a little anxious about my cancer.
- ☐ I am anxious about my cancer.
- ☐ I am very anxious about my cancer.

Which issues have been the most important to you <u>during the past 7 days?</u>
Check ☑ up to 3 boxes.

☐ Pain	☐ Swallowing	☐ Taste
☐ Appearance	☐ Chewing	☐ Saliva
☐ Activity	☐ Speech	☐ Mood
☐ Recreation	☐ Shoulder	☐ Anxiety

©University of Washington, 1999

GENERAL QUESTIONS

Compared to the month before you developed cancer, how would you rate your health-related quality of life? (check one box: ☑)

- ☐ Much better
- ☐ Somewhat better
- ☐ About the same
- ☐ Somewhat worse
- ☐ Much worse

In general, would you say your **health-related quality of life** during the past 7 days has been: (check one box: ☑)

- ☐ Outstanding
- ☐ Very good
- ☐ Good
- ☐ Fair
- ☐ Poor
- ☐ Very poor

Overall quality of life includes not only physical and mental health, but also many other factors, such as family, friends, spirituality, or personal leisure activities that are important to your enjoyment of life. Considering everything in your life that contributes to your personal well-being, rate your **overall quality of life** during the past 7 days. (check one box: ☑)

- ☐ Outstanding
- ☐ Very good
- ☐ Good
- ☐ Fair
- ☐ Poor
- ☐ Very poor

Please describe any other issues (medical or nonmedical) that are important to your quality of life and have not been adequately addressed by our questions (you may attach additional sheets if needed).

©University of Washington, 1999
